# Isotemporal substitution effect of 24-hour movement behavior on the mental health of Chinese preschool children

**DOI:** 10.3389/fpubh.2024.1288262

**Published:** 2024-03-15

**Authors:** Fang Li, Long Yin, Wanhong Luo, Zan Gao, Suryeon Ryu, Mingyun Sun, Pan Liu, Zongyu Yang

**Affiliations:** ^1^School of Physical Education, Hunan First Normal University, Changsha, China; ^2^College of Physical Education, Hunan Normal University, Changsha, China; ^3^Department of Kinesiology, Recreation, and Sport Studies, The University of Tennessee, Knoxville, TN, United States; ^4^School of Kinesiology, University of Minnesota-Twin Cities, Minneapolis, MN, United States; ^5^School of Mathematics and Statistics, Hengyang Normal University, Hengyang, China; ^6^School of Physical Education, Hunan University of Technology, Zhuzhou, China; ^7^School of Physical Education and Health Sciences, Guangxi University for Nationalities, Nanning, China

**Keywords:** isotemporal substitution, 24-h movement behavior, mental health, screen-based sedentary behavior, non-screen-based sedentary behavior

## Abstract

The 24-h movement behavior of preschoolers comprises a spectrum of activities, including moderate-to-vigorous intensity physical activity (MVPA), light-intensity physical activity (LPA), screen-based sedentary behavior (SCSB), non-screen-based sedentary behavior (NSCSB), and sleep. While previous research has shed light on the link between movement behaviors and children’s mental health, the specific impacts on the unique demographic of Chinese preschoolers remain underexplored. This study significantly contributes to the literature by exploring how 24-h movement behavior affects the mental health of preschoolers in a Chinese context. The study involved205 Chinese preschool children (117 boys and 88 girls) between the ages of 3 and 6 years wore accelerometers to measure their LPA, MVPA, and sedentary behavior (SB), while their parents reported the time spent on sleep and SCSB. The parents also completed the Strength and Difficulties Questionnaire to assess their children’s mental health. The study used compositional regression and isotemporal substitution models to examine the relationship between the various components of 24-h movement behavior and mental health. The results showed that greater NCSSB compared to MVPA, LPA, sleep, and SCSB was associated with good prosocial behavior and lower scores on externalizing problems. This highlights the potential of NSCSB as a beneficial component in the daily routine of preschoolers for fostering mental well-being. Replacing 15 min of sleep and SCSB with 15 min of NSCSB was associated with a decrease of 0.24 and 0.15 units, respectively, in externalizing problems. Reallocating 15 min of sleep to NSCSB was linked to an increase of 0.11 units in prosocial behavior. There were no significant substitution effects between LPA and MVPA time with any other movement behavior on prosocial behavior and externalizing problems. Given the positive associations observed, further longitudinal studies are necessary to explore the link between 24-h movement behavior and mental health in preschool children.

## Introduction

Psychosocial well-being is an essential aspect of health, according to the World Health Organization (WHO) (1). The relationship between mental and physical well-being is bidirectional, and mental health has a significant impact on physical health and vice versa, as emphasized by the tenet “There is no health without mental health” (2). A myriad of factors impact the psychological welfare of children. These encompass television watching duration, sleep, and physical activity (PA), with each factor playing a unique role in shaping mental health outcomes.

Moderate-to-vigorous intensity physical activity (MVPA) has consistently been associated with a range of physiological and psychological benefits in children and adolescents (3, 4). Additionally, other studies have shown the benefits of light-intensity physical activity (LPA) and total physical activity (TPA) on mental health (5). Conversely, inadequate sleep duration has been related to adverse health outcomes (6, 7), while extended screen time and specific computer usage patterns have been linked to negative mental health outcomes (8, 9). Different types of sedentary behavior (SB) yield diverse health implications (10, 11). In this manuscript, Screen-Based Sedentary Behavior (SCSB) refers to sedentary activities involving electronic screen devices, such as watching television, using computers, or smartphones. Studies have linked SCSB to various health risks (8, 12, 13). Conversely, non-Screen-Based Sedentary Behavior (NSCSB) encompasses sedentary activities that do not involve electronic screens, such as reading or storytelling with parents. These activities have been associated with cognitive development and mental health (12).

However, previous studies have focused only on the impact of PA or a specific behavior on mental health among children. In recent years, several scholars have stressed the need to comprehensively consider the combined effect of multiple behaviors, including PA, sleep, and SB (14–16). To achieve optimal health in humans, Pedisic has proposed an “activity balance model” that advocates for balancing PA, sleep, and SB for 24 h a day (17).

Consequently, researchers have begun examining the combined effects of 24-h movement behaviors on individuals’ health and academic achievement (18–20). Studies have shown associations between health indicators and specific combinations of movement behaviors (21–24). However, traditional linear regression analysis cannot fully interpret an individual’s 24-h movement due to statistical limitations that conflict with the nature of the time constitution. Specifically, traditional methods do not adequately account for the “unit-sum constraint in 24-h movement behaviors, which means that the increase in time spent on one activity necessitates a decrease in another, making these behaviors interdependent (25). This inherent characteristic of time use data makes them compositional in nature, where analyzing the parts individually without considering their relative proportions can lead to misleading interpretations.

To overcome this limitation, Compositional Data Analysis (CoDA) has emerged as a statistical method in the health promotion field to analyze an individual’s 24-h movement behavior (26). CoDA involves expressing the time spent in different movement behaviors during a fixed period in relative terms as a set of isometric log-ratio coordinates (27). Additionally, Chastin et al. proposed a compositional isotemporal substitution model, which is particularly useful for exploring the theoretical effects of substituting one type of time-use behavior for another, providing insights into how reallocations of time can influence health outcomes (25). Using this technique, researchers have examined the relationships among reallocations of time use, health, and well-being. For example, reallocations of different movement compositions, such as from MVPA to sleep, SB, or LPA, or from sleep, SB, or LPA to MVPA, has shown considerable impact on health outcomes in various populations, including children, adults, and older adults (28–30).

Although numerous studies have investigated the relationships between time-use behavior and physical and mental health across different age groups, there is a limited number of research that have employed the compositional isotemporal substitution method to explore the link between 24-h movement behavior and health in preschool children (31–33). Additionally, studies like Feng et al. have primarily focused on the effectiveness of parent-centered interventions in modifying Chinese preschoolers’ movement behaviors (34), but they do not delve into the direct impact of these behavior modifications on the children’s mental health. Moreover, only one study has specifically focused on the mental health outcomes of substituting one type of movement behavior with another in Chinese preschoolers (35). This research gap is particularly significant considering the unique socio-cultural and environmental context of China, such as rapid urbanization, the emphasis on early academic achievement, and lifestyle, all potentially influencing preschoolers’ mental health and development. While our study focuses on Chinese preschool children, the insights gained could be valuable for understanding similar dynamics in different cultural contexts or populations.

Using the compositional isotemporal substitution method to explore mental health outcomes in Chinese preschoolers is crucial to inform targeted health policies and interventions in China’s unique socio-cultural context. Our study’s findings could offer vital insights for optimizing mental health in early childhood, particularly in balancing educational pressures and PA in China. Therefore, the primary objective of this study is to use the compositional isotemporal substitution method to investigate the effects of reducing time spent in one behavior while increasing time spent in another behavior on Chinese preschool children’s mental health outcomes, amidst these unique socio-cultural and environmental settings. Furthermore, the methodological approach and results of our research may provide a framework for broader applications in child mental health studies, contributing to a more comprehensive understanding of the impact of daily behaviors on young children’s mental health globally. Our hypotheses are (a) individuals who spend a higher proportion of their total sedentary time in NSCSB, as opposed to SCSB, will demonstrate significantly better mental health outcomes and (b) substituting a fixed duration of one movement behavior for another (for example, replacing 15 min per day of SCSB with an equivalent duration of NSCSB) will lead to changes in mental health outcomes, which could either be improvements or a decrease, depending on the nature of the substitution.

## Materials and method

### Study design and participants

This cross-sectional study focused on preschool children between the ages of 3 and 6, of both sexes, who were enrolled in early childhood education and care services (ECECs) licensed by the Hengyang Municipal Bureau of Education, located in the south–central region of Hunan province in China. The Preschool education zone in Hengyang City Urban Area is organized into 5 sectors, housing 90 ECECs. In order to ensure a representative sample across the city’s diverse educational sectors, 5 institutions were strategically selected for this study. One ECEC was randomly selected from each of the 5 sectors, with 3 different level classes (Nursery Class, Middle Class, Senior Class) selected in each ECEC. Collectively, a total of 306 registered preschool children were invited to participate in this study. Prior to the study, the researchers provided all parents of preschoolers with a written consent form that outlined the aim and procedures of the study. The form was distributed to obtain permission from the parents to allow their children to participate. There were thirty-eight households that did not provide written consent, and an additional thirty children did not attend the assessment days. During the assessments, thirty-three children did not provide complete data on PA, sleep duration, screen time, SB, physical fitness, and psychosocial functioning. Of the final sample, there were 117 boys and 88 girls (*M* = 4.8 years old; SD = 0.51). The study protocol was approved by the Institutional Review Board of Hengyang Normal University in 2021 (no. 2021003).

## Measures

### PA and SB

Preschoolers’ PA and SB were objectively measured using an accelerometer (Actigraph, model wGT3X-BT, United States). The device was affixed to the right hip of each participant for seven consecutive days, except during bathing, swimming, and sleeping periods. In accordance with Choi et al.’s wear-time algorithm (36), valid accelerometer data was collected from participants who wore the device for at least 8 h per day, for a minimum of three valid days (including two weekdays and one weekend). Childcare staff and parents were provided with both verbal and written instructions on how to wear and remove the accelerometer device, and were advised to monitor the device’s usage and record the times in a wear log. Data was collected in 15-s epochs using ActiLife software (ActiGraph Corps., Pensacola, FL, United States; Version 6.13.3), and Butte’s et al. (36) cut points were used to classify movement behavior into SB (≤ 239 counts/epoch), LPA (240–2,119 counts/epoch), MPA (2120–4,449 counts/epoch), and VPA (≥ 4,450 counts/epoch) (37).

SCSB was assessed by asking two questions: “How many hours does your child spend watching TV, using the computer, using a smartphone, or playing videogames on weekdays?” and “How many hours does your child spend watching TV, using the computer, using a smartphone, or playing videogames on weekends?.” Daily SCSB time was calculated as follows: [(SCSB on weekdays × 5) + (SCSB on weekends × 2)]/7. The daily NSCSB (e.g., homework, reading) was calculated as follows: (daily SB time—daily SCSB).

The parents of preschoolers were asked to report their children’s daily sleep with a questionnaire, which included two questions about wake-up times and bedtime on both weekdays and weekends to assess nighttime sleep and other two question about daytime sleep on both weekdays and weekends (38). The daily sleep was calculated as follows: [((daytime sleep on weekdays*5 + daytime sleep on weekends*2) + (nighttime sleep on weekdays*5 + nighttime sleep on weekends*2))/7].

### Mental health

The children’s mental health outcomes, including emotional symptoms, conduct problems, hyperactivity/inattention, peer relationships problems, and prosocial behavior, were measured using the Strength and Difficulties Questionnaire (SDQ) (39, 40). The questionnaire consists of 25 items rated on a 3-point scale, completed by parents. Each subscale with five items generated a score, and the total difficulties scale was derived by summing up the scores across the four problems subscale (emotional symptoms, conduct problems, hyperactivity/inattention, and peer relationships problems). However, in our study, we adopted the 3-subscale model of SDQ (internalizing problems [INTER; the sum of emotional symptoms and peer relationships problems subscales], externalizing problems [EXTER; the sum of conduct problems and hyperactivity/inattention subscales], and prosocial behavior [PRO]) following the recommendations for general and low-risk population studies (41, 42). This model was specifically chosen for its relevance and efficiency in assessing the mental health aspects most pertinent to our research demographic, ensuring a focused and effective analysis. Higher scores indicate more significant problems with internalizing problems and externalizing problems. However, for prosocial behavior, higher score indicates better psychosocial functioning.

### Demographic characteristics

After obtaining parental consent, the parents of the children completed a demographic questionnaire which included the child’s age and gender, as well as the annual family income and the education level of the parent. The socioeconomic status (SES) index was calculated using principal component analysis and classified into three grades: low, medium, and high. The participants’ height and weight were measured by the kindergarten physician, and we calculated the body mass index (BMI) by multiplying the weight and height (weight/height2 [kg/m2]).

To ensure the credibility of our questionnaire, we implemented a test–retest method, re-administering the questionnaire to the same participants after a two-week interval. The obtained reliability coefficient was 0.88, which is considered highly reliable.

### Statistical analysis

The data were analyzed using the packages of composition in R v. 4.1.3 (R Core Team, Vienna, Austria) and RStudio v. 4.1.3 (RStudio Team, Boston, MA) (31, 43). In our study, we applied the isometric log-ratio (ilr) transformation to compositional data, converting each component into logarithmic ratios against the geometric mean of other components. This approach effectively normalizes the data, overcoming the constant sum constraint and facilitating standard statistical analysis. Firstly, descriptive statistics were used to describe the distribution characteristics of all demographic and outcome variables. The geometric means and a variation matrix were calculated to describe the movement behavior composition. Then, we used linear regression to explore the correlation between mental health and the movement behavior composition, expressed as isometric log ratios (ilr). These models were adjusted for age, sex, BMI, and SES. The specific multiple linear regression models are:


$$\begin{array}{l} E\left(Y|Z\right)=\beta_{0}+\beta_{1}Z_{1}+\beta_{2}Z_{2}+\beta_{3}Z_{3}+\cdots +\beta_{d}Z_{d-1}\\ \;+\mathrm{age}+\mathrm{sex}+BMI+SES \end{array}$$

$$Z_{i}=\frac{\sqrt{d-i}}{\sqrt{d-i+1}}\ln\left(\frac{b_{i}}{\sqrt[{d-i}]{\prod_{j=i+1}^{d}b_{j}}}\right)$$

d represents the number of components in the composition data, where i = 1,2,3,⋯, d-1, $Z_{i}$ represents the ilr transformation variable, and $b_{i}$ represents the corresponding component data. Y represents the dependent variable. Finally, we used isotemporal substitution models to estimate the substitution impact of replacing one movement behavior for an equal duration. Findings will be presented in tables, showing 15-min reallocations between behaviors like sleep, SCSB, NSCSB, LPA, and MVPA, along with trend charts for a visual interpretation of these changes.

## Results

### Descriptive characteristics

The descriptive statistics for key study variables are displayed in Table 1. On average, participants engaged in 106.6 min (7.5%) of SCSB, 413.6 min (29.2%) of NSCSB, 209.4 min (14.8%) of LPA, 48.6 min (3.4%) of MVPA, and 636.8 min (45%) of sleep per day. There were no significant differences in the geometric percentages and component percentages.

**Table 1:** Descriptive statistical analysis of key variable.

	SCSB	NSCSB	LPA	MVPA	SP	INTER	EXTER	PRO
min/day-mean (Std. deviation)	106.6 (63.1)	413.6 (77.3)	209.4 (30.8)	48.6 (14.5)	636.8 (56.2)	5.8 (2.3)	6.0 (2.1)	6.7 (1.7)
percentage%	7.5	29.2	14.8	3.4	45.0			
Composition mean percentage%	0.08 8.0	0.289 28.9	0.147 14.7	0.033 3.3	0.451 45.1			

LPA: light physical activity; MVPA: moderate-to-vigorous physical activity; SP: sleep time; SCSB: screen-based sedentary behavior time; NSCSB: non-screen-based sedentary behavior time. PRO: Prosocial Behaviors; INTER: internalizing problems; EXTER: externalizing problem.

All pair-wise log-ratio variances are summarized in the variation matrix (Table 2). When the ratios are close to zero, the time spent in each behavior is highly proportional. For our sample, the highest log-ratio variance included all SCSB, suggesting that SCSB is the least co-dependent on the other behaviors.

**Table 2:** Variation matrix of 24-h movement behavior.

	LPA	MVPA	SP	SCSB	NSCSB
LPA	0.00	0.06	0.04	0.58	0.07
MVPA	0.06	0.00	0.10	0.62	0.15
SP	0.04	0.10	0.00	0.53	0.06
SCSB	0.58	0.62	0.53	0.00	0.79
NSCSB	0.07	0.15	0.06	0.79	0.00

LPA: light physical activity; MVPA: moderate-to-vigorous physical activity; SP: sleep time; SCSB: screen-based sedentary behavior time; NSCSB: non-screen-based sedentary behavior time.

### Association between 24-h movement behavior compositions and mental health

The first three-factor model of SDQ with 25 items did not present adequate adjustment indexes (RMSEA = 0.07, CFI = 0.69, TLI = 0.66). Therefore, we revised the model to better fit our data, excluded the items with low loadings (6, 11, 23, 7, 12, 22, 21, 25) based on the modification indices. We then added one item onto the prosocial factor from peer relationships problems subscales. The second structural model demonstrated adequate adjustment indexes in terms of RMSEA (0.04) [0.03–0.06], CFI (0.93), and TLI (0.91).

The result of the compositional regression model was displayed in Table 3 and shows that movement behavior composition was significantly related to prosocial behaviors (R^2^ = 0.04, *p* < 0.05) and externalizing problem (R^2^ = 0.24, *p* < 0.01), but not internalizing problems (R^2^ = 0.006, *p* > 0.05). Specifically, there was a noteworthy negative association between externalizing problems and NSCSB compositions, and a positive association between prosocial behavior and NSCSB compositions. A significant negative association was found when sleep predicted prosocial behavior (*p* = 0.01), and there were significant positive associations between the prosocial behavior and the compositions of NSCSB (*p* < 0.01), whereas null associations were observed for the other relationships.

**Table 3:** The association between 24-h movement behavior compositions with mental health.

	SCSB		NSCSB		SP		LPA		MVPA		Model
	$\beta_{1}^{\left(1\right)}$ [95% CI]	*p*	$\beta_{1}^{\left(2\right)}$ [95% CI]	*p*	$\beta_{1}^{\left(3\right)}$ [95% CI]	*p*	$\beta_{1}^{\left(4\right)}$ [95% CI]	*p*	$\beta_{1}^{\left(5\right)}$ [95% CI]	*p*	P (R^2^)
PRO	0.30 [−0.2,0.8]	0.22	2.57 [1.1,4.0]	**<0.01**	−4.25 [−7.6,-0.9]	**0.01**	−0.41 [−2.5,1.7]	0.7	−0.31 [−1.5,0.9]	0.60	**<0.05 (0.04)**
INTER	−0.06 [−0.7,0.6]	0.86	−0.08 [−2.0,1.8]	0.94	0.01 [−4.2,4.3]	0.99	0.1 [−2.6,2.8]	0.93	−0.88 [−2.4,0.6]	0.25	>0.05 (0.006)
EXTER	0.60 [−0.02,1.2]	0.06	−4.0 [−5.8, −2.2]	**<0.01**	0.07 [−3.6,5.0]	0.75	0.04 [−2.7,2.7]	0.97	−0.85 [−2.3,0.6]	0.26	**<0.01 (0.24)**

LPA: light physical activity; MVPA: moderate-to-vigorous physical activity; SP: sleep time; SCSB: screen sedentary behavior time; NSCSB: non-screen sedentary behavior time. PRO: prosocial behaviors; INTER: Internalizing problems; EXTER: externalizing problem. All models are adjusted for age, sex, and SES. Statistically significant associations (*p* < 0.05) are highlighted in bold.

### Predictions for reallocation of time

Based on the 95% confidence interval (CI), compositional isotemporal substitution was carried out for prosocial behavior and externalizing problem outcome measures. The results of the reallocating 15 min between movement behavior are displayed in Table 4. Replacing 15 min of SCSB time with NSCSB time was associated with a 0.24 unit decrease in the externalizing problem, while replacing 15 min of NSCSB time with SCSB was associated with a 0.22 unit increase in the externalizing problem. Substituting sleep with NSCSB time in 15 min was associated with a 0.11 unit increase in prosocial behavior and a 0.15 unit decrease in externalizing problems. Replacing 15 min of NSCSB time with sleep was associated with a 0.11 unit decrease in prosocial behavior and a 0.16 unit increase in the externalizing problems. No significant substitution effects were found between LPA and MVPA time with any other movement behavior on prosocial behavior and externalizing problems. However, these findings are preliminary and highlight the need for further research to understand the causal relationships.

**Table 4:** Estimated difference in mental health for 15-min isotemporal substitution between 24-h movement behaviors.

Add	Remove	PRO (95% CI)	EXTER (95% CI)
SCSB	SP	0.07 (−0.12,0.16)	0.07 (−0.04,0.18)
SCSB	LPA	0.05 (−0.10,0.20)	0.07 (−0.12,0.26)
SCSB	MVPA	0.14 (−0.25,0.53)	0.25 (−0.24,0.75)
SCSB	NSCSB	−0.04 (−0.10,0.01)	0.22* (0.16,0.29)
LPA	SP	−0.02 (−0.12,0.15)	0.00 (−0.18,0.17)
LPA	MVPA	0.08 (−0.39,0.56)	0.18 (−0.42,0.79)
LPA	SCSB	−0.06 (−0.21,0.09)	−0.09 (−0.28,0.10)
LPA	NSCSB	−0.09 (−0.24,0.05)	0.15 (−0.03,0.33)
MVPA	SP	−0.04 (−0.3,0.25)	−0.15 (−0.51,0.22)
MVPA	LPA	−0.05 (−0.43,0.32)	−0.15 (−0.63,0.34)
MVPA	SCSB	−0.11 (−0.40,0.17)	−0.24 (−0.60,0.13)
MVPA	NSCSB	−0.15 (−0.43,0.13)	0.01 (−0.35,0.36)
SP	LPA	−0.02 (−0.16,0.13)	0.00 (−0.18,0.18)
SP	MVPA	0.07 (−0.33,0.46)	0.19 (−0.32,0.69)
SP	SCSB	−0.07 (−0.17,0.02)	−0.09 (−0.21,0.03)
SP	NSCSB	−0.11* (−0.18, −0.04)	0.16* (0.06,0.25)
NSCSB	SP	0.11* (0.04–0.18)	−0.15* (−0.24,−0.06)
NSCSB	LPA	0.09 (−0.05,0.24)	−0.15 (−0.34,0.04)
NSCSB	MVPA	0.18 (−0.21,0.56)	0.03 (−0.46,0.53)
NSCSB	SCSB	0.04 (−0.02,0.09)	−0.24*(−0.32,−0.16)

LPA: light physical activity; MVPA: moderate-to-vigorous physical activity; SP: sleep time; SCSB: screen-based sedentary behavior time; NSCSB: non-screen-based sedentary behavior time. PRO: prosocial behaviors; EXTER: externalizing problem. All models are adjusted for age, sex, BMI, and SES. Statistically significant associations at the 95% confidence interval (CI) level (*p* < 0.05) are highlighted in bold.

**Figure 1:**
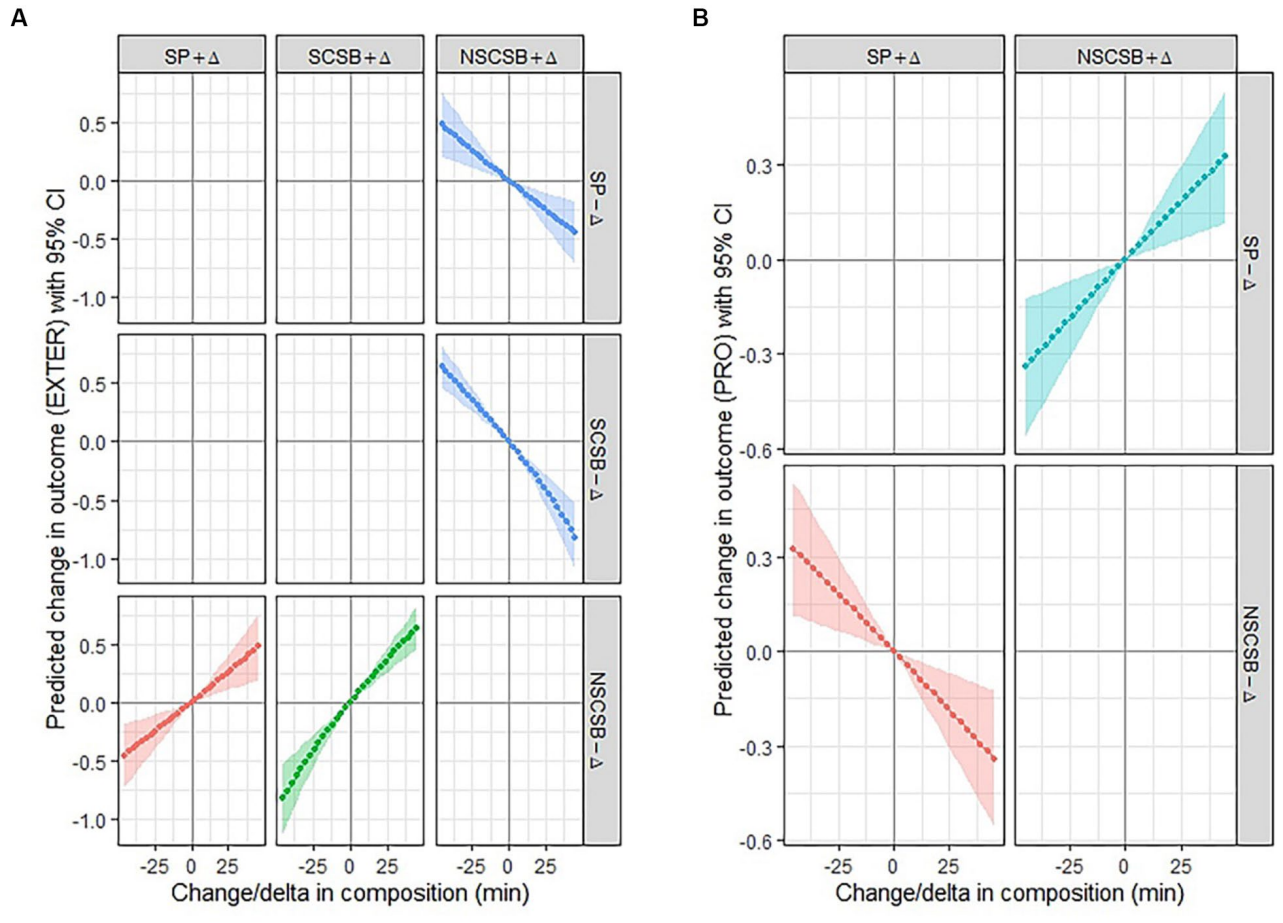
**(A)** The changing trend in externalizing problems after isotemporal substitutions. **(B)** The changing trend in prosocial behavior after isotemporal substitutions. Analyses controlled for age, sex, BMI and SES, PRO indicates prosocial behavior, SCSB: screen-based sedentary behavior time; NSCSB: non-screen-based sedentary behavior time; SP: sleep time.

The estimated differences in the externalizing problem demonstrated a linear association with increasing increments of time reallocated between sleep time and NSCSB time (Figure 1A). Similarly, Figure 1B displayed a linear association between the reallocated NSCSB time, SCSB time, and sleep time, in relation to the estimated differences in prosocial behavior.

## Discussion

The purpose of this investigation is to utilize compositional data analysis to explore the connections between 24-h movement behavior patterns and mental health in Chinese preschoolers. Additionally, this study aims to examine the effects of isotemporal reallocation of 24-h movement behavior on mental health outcomes. The results of this research reveal that a substantial amount of time for Chinese preschoolers is spent in SB (36.7%), with only a small proportion of time allocated for MVPA (3.4%). These findings are consistent with earlier studies on PA and SB in preschool-aged children (33, 44). However, previous research suggests that the amount of time devoted to PA and SB varied greatly, and results were inconsistent depending on the measurement techniques employed in each study (45, 46). Therefore, it is recommended that objective measurement methods, such as accelerometry, be used to minimize errors.

Previous research has consistently reported a positive relationship between the total amount of SB and externalizing problems (47), as well as internalizing problems (48). Moreover, a negative association has been observed between total SB and prosocial behavior (48). However, our study provides a nuanced understanding of these relationships by examining NSCSB and SCSB separately. Specifically, NSCSB activities like storytelling, drawing, or parent–child reading, may contribute positively to mental health by enhancing social–emotional development. Our findings revealed that NSCSB was negatively associated with externalizing problems, while SCSB had no significant impact on externalizing problems. Furthermore, a systematic review conducted by Poitras and colleagues suggested that NSCSB activities, such as storytelling and drawing, may facilitate the social–emotional development of young children (12). Additionally, we observed that NSCSB (relative to other behaviors) was positively associated with prosocial behavior in Chinese preschoolers, while SCSB was unlikely to affect prosocial behavior. Bourke’s study also showed that spending more time being sedentary relative to other movement behaviors during waking hours was associated with better psychosocial functioning (31). Nevertheless, in contrast to the influence of specific behaviors on mental health (49), when considering compositional data, there was no notable connection observed between internalizing problems and the arrangement of movement behaviors. A study employing compositional data analysis in Canada arrived at analogous findings (50). These contrasting findings highlight the complexity of the relationships between types of SB and mental health, underscoring the need for a nuanced approach in interventions. It is important to not only encourage NSCSB but also consider the role of sleep behavior within the 24-h movement behavior framework, as sleep and mental health are closely linked, and adequate sleep duration has been associated with better mental health (6, 51). However, research also suggests that both too little and too much sleep can have negative impacts on mental health (52). Our study found significant negative associations between the compositions of sleep behavior and prosocial behavior (Table 3). The significant association of sleep behavior with prosocial behavior, combined with the lack of association between PA compositions and mental health, challenges the simplistic binary of ‘active = good’ and ‘sedentary = bad.’ This indicates a more intricate interplay of various behaviors and their cumulative effect on mental health, which future research should explore in greater depth. Considering 24-h movement behavior as a whole, time spent in one behavior is naturally related to time spent in other behaviors, and the impact of time spent in one behavior depends on the rest of the day. Preschoolers spend a relatively large proportion of their day in sleep (636.8 min or 56.8%), which may decrease the time available for other activities, such as social interactions, and negatively affect their prosocial behavior. In addition, there was no association between the compositions of PA (including LPA and MVPA) and mental health, indicating that a small proportion of time spent in MVPA compared to other movement behaviors did not seem to have any effect on mental health. This does not imply that PA is not related to mental health. In fact, PA has been found be beneficial on individuals’ mental health (53, 54). Yet, in line with Brown’s findings, our results yield no association between compositions of PA and mental health (55). These results suggest that the interaction between different types of movement behaviors and their cumulative effect on mental health may be more complex than previously understood. It is important to note that the compositions of PA and PA itself are not the same concept. The time spent engaging in different behaviors is interdependent and limited during the 24-h period (56). Hence, it is plausible that one ‘unhealthy” movement behavior may offset the health benefits of another healthy behavior. Moreover, the relatively limited portion (3.3%) of time dedicated to MVPA, in comparison to various other movement behaviors, might not have effectively countered the plausible adverse impacts stemming from a greater share of time allocated to alternative “unhealthy” movement behaviors. These results are consistent with previous studies (50).

The results of the compositional isotemporal substitution models are shown in Table 4 and Figures 1A,B. These models indicate that substituting 15 min of SCSB with NSCSB is associated with a reduction of 0.24 units in externalizing problems in Chinese preschoolers. Similarly, replacing sleep with NSCSB is also associated with lower externalizing problems, and vice versa. On the other hand, replacing NSCSB with SCSB or sleep leads to an increase in externalizing problems. This finding further supports the unique role of NSCSB in influencing children’s behavioral development and underscores the importance of specific types of activities in shaping mental health outcomes. In particular, replacing different types of SB can effectively decrease preschoolers’ externalizing problems. NSCSB, such as parent–child activities like storytelling, drawing, handwork, and reading together, can help children learn to regulate their emotions and behavior, leading to improved social interactions and adjustments to different situations (12, 57). In contrast, an increase in SCSB, such as watching television or playing video games, has been linked to an increase in externalizing problems, and screen time has been associated with an increased risk of aggressive behavior in preschoolers (58–60). By encouraging more NSCSB activities in preschoolers’ daily routines, parents and educators can play a key role in fostering children’s mental health and social development.

Furthermore, NSCSB derived from sleep may help reduce preschoolers’ externalizing problems. It suggests that activities involving NSCSB, such as interactive play or creative tasks, could be effective in mitigating behavioral issues in young children. It is important to note that NSCSB may moderately reduce the sleep duration in preschoolers based on the 24-h movement behavior framework. However, this suggestion should be treated with caution as adequate sleep has a protective impact on mental health. Our research did not find a significant association between sleep composition and externalizing problems, likely due to the little variation in sleep duration among Chinese preschoolers who mostly meet the 24-h movement guidelines (23). Additionally, we were only able to collect data on sleep duration and not sleep efficiency using questionnaires. Future studies should consider distinguishing between high-quality and lower-quality sleep duration when collecting sleep duration data to improve the accuracy of findings. Practical recommendations for parents and educators might include structuring the day to allow for both adequate sleep and sufficient time for NSCSB, recognizing the importance of each in contributing to a child’s overall mental health and social development.

Preschoolers who engaged in more than 15 min of NSCSB time and replaced it with 15 min of sleep time showed an increase of 0.11 units in prosocial behavior (Table 4; Figure 1B). Conversely, substituting NSCSB time with sleep time resulted in a decrease in prosocial behavior. Previous studies have shown that high levels of SCSB are negatively associated with prosocial behavior, while NSCSB has a positive correlation with social–emotional development (61). Some studies have also suggested that SB is associated with better sleep efficiency (62, 63), which is further positively associated with better emotional and behavioral functioning in preschoolers (64). Therefore, substituting a small amount of NSCSB for an equal amount of sleep time, while ensuring adequate sleep, can promote parent–child interaction and improve preschoolers’ prosocial behavior. This highlights the potential benefits of encouraging activities that promote social interaction and reduce screen time in preschool children’s daily schedules.

The identification that substituting sleep and SCSB with NSCSB has a positive impact on externalizing problems and prosocial behavior among preschoolers holds significant implications for the fields of clinical practice and public health. These findings provide a strong basis for developing targeted interventions and programs that prioritize NSCSB in early childhood settings, aiming to foster healthy behavioral and social development. In summary, our study offers actionable insights for creating nurturing environments that promote balanced and healthy behaviors in preschool children, potentially leading to improved mental health outcomes. By emphasizing the role of NSCSB, and considering its interaction with sleep and PA, we can contribute to the holistic development of children during their formative years. This study employed compositional data analysis and accelerometers to objectively measure PA and SB, and applied isochronous substitution to investigate the relationships between 24-h movement behavior and mental health in Chinese preschoolers. However, the study has several limitations that need to be considered when interpreting the findings. One significant limitation is that the study did not capture the entire 24 h of accelerometer data, potentially affecting the comprehensiveness of our movement behavior data. The data on children’s sleep time, screen time, and mental health were collected through parent proxy, which may lead to biased reporting, and the questionnaires used could not measure sleep efficiency. Furthermore, potential measurement inaccuracies could be linked to the computation of NSCSB, which was determined using [SB time – SCSB time]. Future research should not only focus on exploring the balance between adequate sleep and other activities, employing direct methods to measure sleep efficiency and NSCSB, but also develop detailed strategies and guidelines for integrating NSCSB promotion into public health initiatives and clinical practices, as this comprehensive approach will significantly contribute to our understanding of their collective and individual impacts on children’s mental health and overall development. Additionally, since the study employed a cross-sectional design, it was not possible to establish causality. Future research should focus on longitudinal studies to better understand the causative relationships between movement behaviors and mental health outcomes in children. Also, family structure and media exposure at home may affect children’s movement behavior (65). Yet, these factors were not assessed in the present study. Future study may also consider the effects from these environmental correlates. Lastly, this study was conducted in a middle-large city in a South-central region of China, which limits the generalizability of the results to other regions or populations. Subsequent research should include participants from a variety of geographic and socio-economic backgrounds, thereby enhancing the representativeness and applicability of the findings.

## Conclusion

The findings of this research indicate that the 24-h movement behavior composition of Chinese preschoolers is linked to their mental health. This study uniquely contributes to the field by highlighting the differential impacts of NSCSB and sleep on the mental health of preschoolers, a previously underexplored area. Reallocating time between these behaviors suggests that substituting NSCSB for sleep and SCSB may result in negative externalizing problems, while increasing NSCSB engagement at the expense of sleep could enhance prosocial behavior and provide adequate sleep for preschoolers. In conclusion, this research not only contributes significantly to the growing body of knowledge in child development and mental health but also lays the groundwork for future research and practical interventions. Our results advocate for the importance of optimizing movement behavior composition in early childhood to improve mental health outcomes, paving the way for longitudinal studies that can provide more comprehensive insights into this relationship.

## Data availability statement

The raw data supporting the conclusions of this article will be made available by the authors, without undue reservation.

## Ethics statement

The studies involving humans were approved by Hengyang Normal University Institutional Review Board (study number: 2021003). The studies were conducted in accordance with the local legislation and institutional requirements. Written informed consent for participation in this study was provided by the participants’ legal guardians/next of kin.

## Author contributions

FL: Funding acquisition, Writing – original draft. LY: Conceptualization, Funding acquisition, Writing – original draft. WL: Data curation, Investigation, Writing – review & editing. ZG: Writing – review & editing. SR: Writing – review & editing. MS: Data curation, Methodology, Writing – review & editing, PL: Data curation, Investigation, Writing – review & editing. ZY: Data curation, Investigation, Resources, Writing – review & editing.
